# Genetic Evaluation of Monthly Test-Day Milk Yields of Jersey Crossbred Cattle Under Farmers’ Production System in Tamil Nadu, India

**DOI:** 10.3390/ani14213152

**Published:** 2024-11-02

**Authors:** Dhanukodialagar Kasiviswanathan, Palaniappan Devendran, Ragothaman Venkataramanan, Subramanian Meenakshisundaram, Ganesamoorthy Senthilkumar, Sunday O. Peters

**Affiliations:** 1 Department of Animal Genetics and Breeding, Madras Veterinary College, Tamil Nadu Veterinary and Animal Sciences University (TANUVAS), Chennai 600 007, Tamil Nadu, India; drkasi.vet@gmail.com; 2 Bioinformatics Centre, Madras Veterinary College, Tamil Nadu Veterinary and Animal Sciences University (TANUVAS), Chennai 600 007, Tamil Nadu, India; 3 Office of the Vice-Chancellor, Tamil Nadu Veterinary and Animal Sciences University (TANUVAS), Chennai 600 051, Tamil Nadu, India; venkyvet@gmail.com; 4 Directorate of Centre for Animal Production Studies, Tamil Nadu Veterinary and Animal Sciences University (TANUVAS), Chennai 600 051, Tamil Nadu, India; dcaps@tanuvas.org.in; 5 College of Food and Dairy Technology, Tamil Nadu Veterinary and Animal Sciences University (TANUVAS), Chennai 600 052, Tamil Nadu, India; senthilkumargtanuvas@gmail.com; 6 Department of Animal Science, Berry College, 2277 Mount Berry Highway, Mount Berry, GA 30149, USA

**Keywords:** Jersey crossbred cattle, genetic correlation, heritability, random regression, REML, test-day milk yield

## Abstract

Tamil Nadu, a state in southern India, is bereft of any native dairy or dual-purpose cattle breeds, and the state depends chiefly on crossbred cattle for milk production. Jersey crossbred cattle account for 90% of the state’s milk production. This genetic evaluation study aimed to assess milk production traits, including test-day milk yields (TDMYs), total lactation milk yield (TMY), and 305-day milk yield (305MY), in these cattle reared under farmers’ conditions. Data on monthly TDMYs (TDMY1 to TDMY10) of Jersey crossbred cows (*n* = 75,627) reared by the farmers over 11 years (2012–2022) were collected for evaluation. The influence of non-genetic factors viz., location (agroclimatic zones), period and season of calving, and parity on TDMYs, TMY and 305MY were assessed, and the variance and covariance components for genetic parameters (heritability and genetic correlation) of the traits were estimated by the method of restricted maximum likelihood (REML) under a multivariate animal model and by a random regression model (RRM). The overall means of various TDMYs ranged from 4.98 (TDMY10) to 9.95 kg (TDMY2), and for TMY and 305MY, the means were 2480.33 and 2393.71 kg, respectively. The heritabilities estimated by the multivariate animal model for TDMYs were moderate and ranged from 0.26 ± 0.02 to 0.37 ± 0.02 and the estimates were less variable throughout lactation, while the estimates obtained by RRM were medium to high and ranged between 0.29 ± 0.01 and 0.67 ± 0.02 for milk yield on day 5 to 305 (DIM 5–DIM 305). The heritabilities estimated for TMY and 305MY were 0.43 ± 0.02 and 0.43 ± 0.02, respectively. The estimates of heritability for mid-lactation yields were comparatively less than those at the beginning and the end of lactation. The moderate to high estimates of heritabilities for TDMYs, TMY, and 305MY obtained in the present study offer ample scope for improving milk production through selective breeding. Genetic correlations between TDMYs estimated by the multivariate animal model were positive and high, with a range between 0.75 ± 0.03 and 0.99 ± 0.00. Considerably higher estimates (0.98–0.99) were observed between adjacent TDMYs showing strong genetic associations. By RRM, genetic correlations estimated between DIMs were positive (except for DIM 5 with DIM 125 to DIM 185 and DIM 125 with DIM 305), and the magnitude of genetic correlation decreased with an increase in the interval between the DIMs. The high genetic correlation observed between the TDMYs (in the early stage of lactation) and total lactation milk yield suggested that these test-day yields could be used favorably for the early selection of cows for milk production, which facilitates reduction in the generation interval and consequently increases the annual genetic gain for the milk production traits.

## Simple Summary

The milk production performance of Jersey crossbred cattle under farmers’ rearing environment in the state of Tamil Nadu, Southern India, is evaluated genetically from the monthly test-day milk yields. Genetic parameters viz., heritability, and genetic correlations were estimated for test-day milk yields, total lactation milk yield, and 305-day milk yield to assess whether the improvement of these traits could be made through selective breeding. The overall means of various test-day milk yields ranged from 4.98 to 9.95 kg, and the mean total lactation milk yield and 305-day milk yield were 2480.33 and 2393.71 kg, respectively. The performance of Jersey crossbred cattle found in the present study indicates the suitability of the crossbreds in the state. The desirable values of estimated genetic parameters observed in the study revealed ample scope for improving milk production traits by selection. Moreover, the early and mid-lactation test-day milk yields could be favorably used for the early selection of cows under the farmers’ rearing environment to improve total milk production.

## 1. Introduction

Tamil Nadu, a state in the southernmost part of India, is bereft of any native dairy or dual-purpose cattle breeds, and the state depends chiefly on crossbred cattle for milk production. Out of the estimated milk production of Tamil Nadu (10.10 million tons as of 2021–2022), about 90 percent was contributed by Jersey crossbred cattle [1]. The breeding policy of the state advocates Jersey as the breed of choice for crossing non-descript cows in the plains of the state with the level of exotic inheritance restricted to 50 percent for improving milk production because of the adaptability of this crossbred and thus, Jersey crossbred cattle are widely reared.

The genetic evaluation of milk production is a demanding task in terms of time and expenses, especially under farmers’ production conditions. As recording daily milk yields of lactation is invariably difficult and expensive under such conditions, test-day milk yield (TDMY) in monthly intervals has been successfully used [2,3] to arrive at total lactation milk yield (TMY) and 305-day milk yield (305MY) in dairy evaluations [4,5]. The available reports also narrate that selection based on early TDMYs is as efficient as on all TDMYs [6,7]. This approach would result in the reduction in generation interval and cost of milk recording and facilitate early culling of animals [8,9].

The genetic evaluation for milk production involves the assessment of the effects of non-genetic factors and the estimation of variance and covariance components for genetic parameters, viz., heritability of the traits and genetic correlation between the traits. The use of accurate models in genetic analysis and precise estimation of genetic parameters contribute to increased efficiency of selection programs for the improvement of milk production. Among the various genetic evaluation methods, the Restricted Maximum Likelihood (REML) method with an animal model is the most commonly employed method to estimate variance components, considering fixed and random effects simultaneously [10,11]. Nevertheless, the Random Regression Model (RRM) is an alternative approach for genetic evaluation of longitudinal traits like milk yield by considering the nature of the data having measures repeated over intervals (TDMYs) in addition to the estimation of both fixed and random effects simultaneously [12]. In RRM, the regression coefficient for each animal is estimated, and the orthogonal Legendre polynomials are used to fit random curves due to their ability to describe the variation along lactation, better convergence, as the control, variable day of lactation is being normalized. When both the additive genetic and permanent environmental components are modeled by Legendre polynomial coefficients over time, the estimate of variance components becomes more accurate [13].

The genetic evaluation of milk production under farmers’ conditions of rearing will be helpful to assess the performance as well as to identify the ways and means to improve its potential. Such genetic evaluation studies on milk production of crossbred Jersey cattle under the farmers’ production system in Tamil Nadu are scanty. Hence, the present study was intended to assess genetically the milk production traits, viz., TDMYs, TMY, and 305MY, by estimating the variance and covariance components and genetic parameters (heritability and genetic correlation) in Jersey crossbred cattle under farmers’ production system in Tamil Nadu.

## 2. Materials and Methods

Records on monthly test-day milk yields of 75,627 Jersey crossbred cows, covering a period of 11 years from 2012 to 2022, were obtained from the field performance recording program of Tamil Nadu Co-operative Milk Producers’ Federation of Tamil Nadu. The crossbreds studied under the farmers’ production system had about 50 percent of Jersey inheritance, with the remaining being from non-descript or a mixture of non-descript and indigenous dairy breeds. The crossbreds were mostly reared under a semi-intensive system and fed with concentrate as well as green and dry fodder besides grazing. The cows were generally inseminated artificially after the second estrus postpartum. The test-day milk yields of the crossbred cows were recorded from several herds and the herd size was invariably small. A test-day milk yield (TDMY) is the quantity of milk produced by a cow over 24 h. The traits considered were ten monthly test-day milk yield records (TDMY1 to TDMY10) obtained between day 5 and day 305 of lactation. The TDMY1 was recorded after five days of lactation and the subsequent TDMYs were recorded at monthly intervals. The milk yields that were less than 50 percent of the previous TDMY and the values beyond three standard deviations (from the mean) for each test-day yield were excluded from the analyses as outliers. The TMY and 305MY were computed from the TDMYs by the Test Interval method as recommended by the International Committee for Animal Recording [14].

### 2.1. Exploratory Analysis

The data were subjected initially to descriptive analysis and then the least-squares method under the General Linear Model (GLM) for the milk-yield traits was carried out by the IBM SPSS Statistics package, version 25.0 [15] to assess the effect of fixed factors. The fixed factors considered were agroclimatic zones (North Eastern, North Western, Western, Cauvery Delta, and Southern zones); period of calving (2012–2015, 2016–2019, and 2020–2022); season of calving [Winter (January–February), Summer (March–May), Southwest monsoon (June–September), and Northeast monsoon (October–December)]; and parity (1st, 2nd, 3rd, 4th, and above). The pedigree details and data structure of performance records of Jersey crossbred cows are given in Table 1 and Table 2, respectively.

### 2.2. Multivariate Animal Model Analysis

Multivariate analysis of TDMYs, total lactation milk yield (TMY), and 305-day milk yield (305MY) was carried out on the records of cows with pedigree information, by fitting an animal model to estimate the variance and covariance components for heritabilities, genetic correlations, and phenotypic correlations. All the fixed non-genetic factors were found to be significant (*p* ≤ 0.05) sources of variation under GLM; hence, all the fixed factors (fixed effects) and the direct animal genetic effect (random effect) were included in the multivariate analysis. The mixed model for single trait analysis used in the present study is given below:

Y_ijk_ = μ + CG_i_ + N_j_ + A_n_ + e_ijk_

where, Y_ijk_ is the k^th^ observation in the i^th^ contemporary group (agroclimatic zone-period-season; 5 zones-3 periods-4 seasons) and the j^th^ parity, μ is the overall mean, CG_i_ is the effect of the i^th^ contemporary group (i = 1 to 60), N_j_ is the effect of the j^th^ parity (j = 1 to 4), A_n_ (*n* = 32,455) is the random animal effect, and e_ijk_ is the residual effect.

The following animal model was used for multivariate analyses of all the traits studied:

$$y_{i}=X_{i}b_{i}+Z_{i}a_{i}+e_{i},$$

In matrix notation:

$$\left[\begin{array}{c} y_{1}\\ :\\ y_{6}\\ :\\ y_{12} \end{array}\right]=\left[\begin{array}{ccccc} X_{1} & .. & 0 & .. & 0\\ : & : & : & : & :\\ 0 & \ldots & X_{6} & \ldots & 0\\ : & : & : & : & :\\ 0 & \ldots & 0 & \ldots & X_{12} \end{array}\right]\left[\begin{array}{c} b_{1}\\ :\\ b_{6}\\ :\\ b_{12} \end{array}\right]+\left[\begin{array}{ccccc} Z_{1} & \ldots & 0 & \ldots & 0\\ : & : & : & : & :\\ 0 & \ldots & Z_{6} & \ldots & 0\\ : & : & : & : & :\\ 0 & \ldots & 0 & \ldots & Z_{12} \end{array}\right]\left[\begin{array}{c} a_{1}\\ :\\ a_{6}\\ :\\ a_{12} \end{array}\right]+\left[\begin{array}{c} e_{1}\\ :\\ e_{6}\\ :\\ e_{12} \end{array}\right]$$

where *y_i_* is the vector of observations for trait *i* [*i* = 1 to 12 (TDMY1 to TDMY10, TMY and 305MY)], *X_i_* is the incidence matrix relating the fixed effects (*b_i_*) to the vector of observations (*y_i_*); *Z_i_* is the incidence matrix relating the vector of direct additive genetic effects (*a_i_*) to *y_i_*, and *e_i_* is the vector of random residual effects assumed to be NID (0, σ^2^_e_) associated with *y_i_*.

### 2.3. Random Regression Model Analysis

For Random Regression Model (RRM) analysis, a single trait linear mixed RRM was applied to TDMY records. A homogenous (constant) residual variance along days in milk was considered on an assumption that residual effects on different days in milk were uncorrelated both within and between cows. In this analysis, the direct genetic (additive) effects and permanent environmental effects were modeled by Legendre polynomials of order three [16], and the milk yield on day 5 to 305 (DIM 5–DIM 305) was estimated. The RRM used in the analysis is described below:

$$Y_{ijmn}=CG_{i}+N_{j}+\sum_{q=1}^{n}\beta_{q}Z_{mnq}+\sum_{q=1}^{n}a_{mq}Z_{mnq}+\sum_{q=1}^{n}pe_{mq}Z_{mnq}+e_{ijmn}$$

where *Y_ijmn_* is the n^th^ observation of cow m of the i^th^ contemporary group (agroclimatic zone-period-season) and the j^th^ parity; *CG_i_* is the effect of the i^th^ contemporary group (i = 1 to 120), *N_j_* is the effect of the j^th^ parity (j = 1 to 6), *β_q_* is the set of q regression coefficients to model average trajectory of the population, *Z_mnq_* is the fixed covariate of Legendre polynomial according to days in milk, *a_mq_* is the set of q additive genetic random regression coefficients for cow m, *pe_mq_* is the set of q permanent environmental random regression coefficients for cow m, and *e_ijmn_* is the random residual effect associated with *Y_ijmn_*.

The variance structure for the random effects of the model was:

$$V\left(\begin{array}{c} a\\ pe\\ e \end{array}\right)=\left(\begin{array}{ccc} G⊗A & 0 & 0\\ 0 & I⊗P & 0\\ 0 & 0 & R \end{array}\right)$$

where *I* is the Identity matrix, *A* is the matrix of additive genetic relationship among the animals, ⊗ is the Kronecker product function, *P* and *G* are covariance matrices for permanent environmental and additive genetic effects, respectively. *R* is the diagonal matrix of the form Iσ^2^_e_ and σ^2^_e_ is the residual variances.

The multivariate analysis and the RRM were carried out using the method of restricted maximum likelihood (REML) in WOMBAT software [17].

## 3. Results

**Figure 1:**
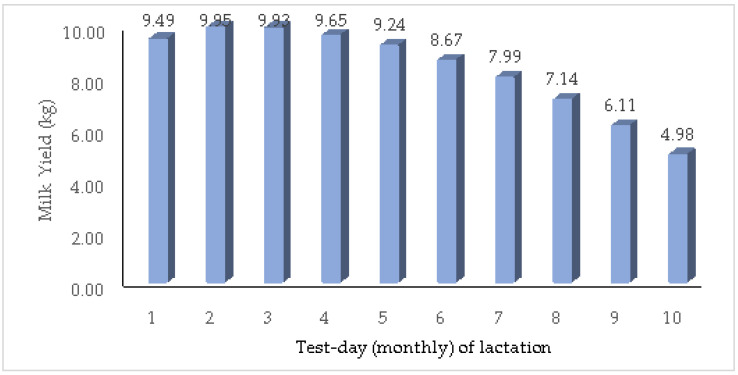
Least-squares mean of test-day milk yields in Jersey crossbred cattle.

The results of the descriptive statistical analysis are presented in Table 3. The mean TDMYs ranged from 4.63 to 9.52 kg and the coefficient of variation ranged from 27.34 to 39.54 percent. The overall least-squares means of various TDMYs ranged from 4.98 in TDMY10 to 9.95 kg in TDMY2 (Figure 1). The trend for TDMYs showed that milk yield was slightly lower at the beginning of lactation, and then it peaked at TDMY2 (9.95 kg) after which the yield displayed a gradual but consistent decline to the end of lactation (Figure 1). The GLM analysis revealed that all the fixed factors under study were significant (*p* ≤ 0.05); therefore, they were included in the multivariate (animal model) and RRM analyses. The overall means estimated for TMY and 305MY in this study were 2480.33 and 2393.71 kg, respectively.

### 3.1. Variance Components

**Figure 2:**
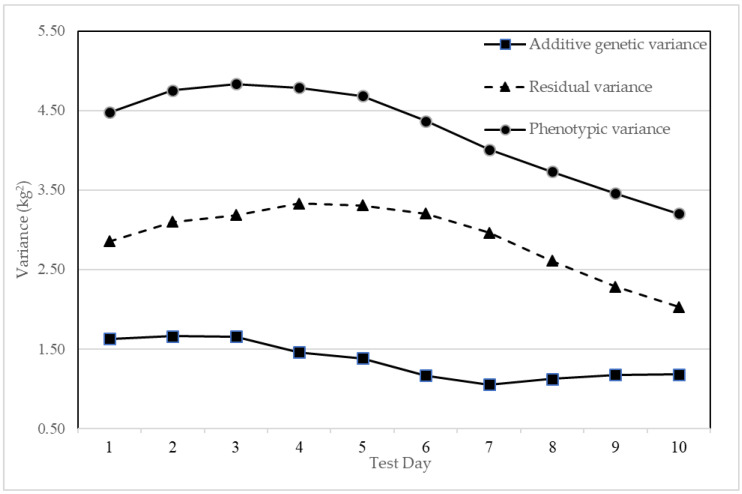
Estimates of variance components for test-day milk yields of Jersey crossbred cattle from multivariate animal model.

The variance components estimated by the multivariate animal model for TDMYs, TMY, and 305MY (Table 4) showed that the estimates of additive genetic variance (V_A_) ranged from 1.05 (TDMY7) to 1.66 (TDMY2) and the variance decreased gradually as lactation advanced (Figure 2). The estimates of residual variances (V_E_) ranged between 2.03 (TDMY10) and 3.33 (TDMY4) and phenotypic variances (V_P_) were dispersed from 3.20 (TDMY10) to 4.83 (TDMY3), indicating higher estimates in the mid-lactation. Both V_E_ and V_P_ exhibited a similar trend of increasing up to TDMY3 and TDMY4 and decreasing towards the end of lactation. Meanwhile, the proportionate contribution of V_A_ to V_P_ gradually decreased for the TDMYs as lactation advanced until TDMY7, and then it increased marginally.

**Figure 3:**
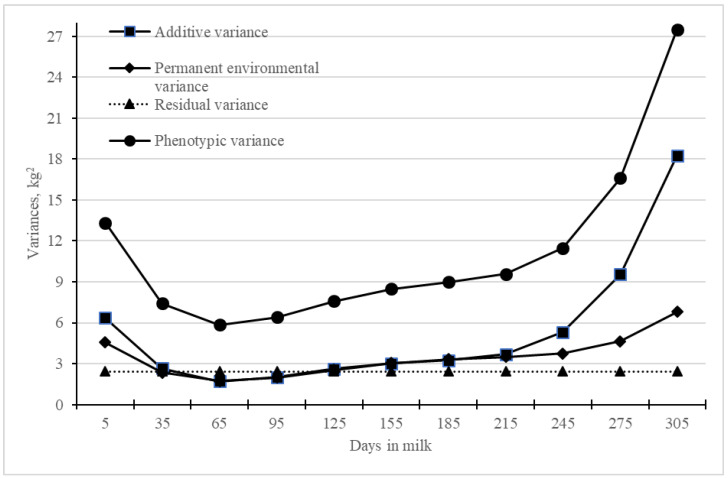
Estimates of variance components for DIMs of Jersey crossbred cattle from random regression model.

In the RRM analysis, the variance components for different DIMs were estimated using the variance–covariance structure among the random regression coefficients and the covariates of the RRM functions (Table 5, Figure 3). The residual variance was assumed constant (homogeneous) throughout lactation. The highest V_A_ was observed for DIM 305 (18.27) and the lowest was observed for DIM 65 (1.69). The permanent environmental variance (V_EP_) was the highest for DIM 305 (6.81) and lowest for DIM 65 (1.73). The magnitude of V_A_ and V_EP_ decreased from DIM 5 to DIM 65 and then increased gradually till the end of lactation (Figure 3). The phenotypic variance (V_P_) was higher at the beginning and end of lactation compared to mid-lactation.

### 3.2. Heritability

**Figure 4:**
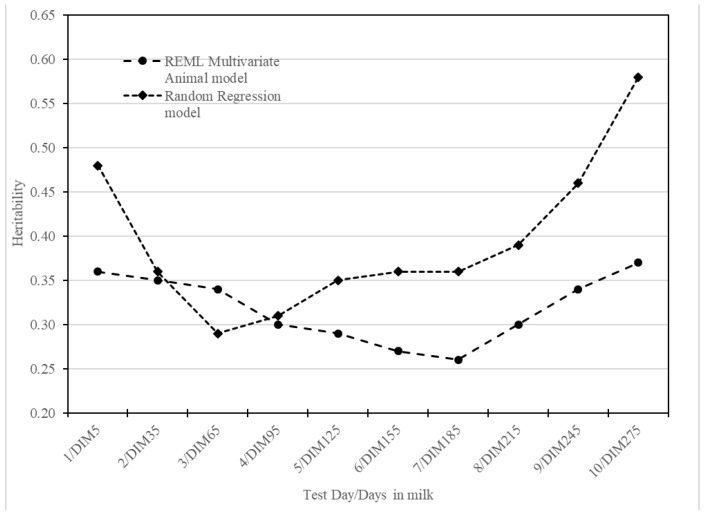
Estimates of heritability for TDMYs from multivariate animal model and DIMs from random regression model of Jersey crossbred cattle.

Heritability estimates, defined as the ratio of additive variance to phenotypic variance, for various TDMYs using the multivariate animal model ranged from 0.26 ± 0.02 to 0.37 ± 0.02 (Table 6). These estimates were moderate in magnitude (Figure 4).

The heritability estimate for both TMY and 305MY was 0.43 ± 0.02 (Table 6). The heritability estimates from RRM (Table 7) were moderate to high for different DIMs (except DIM 65) and ranged from 0.29 ± 0.01 to 0.67 ± 0.02, showed a decreasing trend from DIM 5 to DIM 65 and then gradually increased until the end of lactation (Figure 4).

### 3.3. Genetic and Phenotypic Correlations

Genetic correlations between TDMYs estimated by the multivariate animal model were positive and high, ranging between 0.75 ± 0.03 and 0.99 ± 0.00 (Table 6). Considerably higher estimates were observed between adjacent TDMYs showing strong genetic association. By RRM, genetic correlations estimated between DIMs were positive (except for DIM 5 with DIM 125 to DIM 185 and DIM 125 with DIM 305) with varying magnitudes (Table 7). The phenotypic correlations from the multivariate animal model between different TDMYs were positive and medium to high (except TDMY1 with TDMY10) and ranged from 0.36 ± 0.01 to 0.88 ± 0.00 (Table 6). The phenotypic correlations tended to decrease as the time interval between test-days increased. Positive and moderate to high phenotypic correlations were found for TDMYs with TMY and 305MY (Table 6). The phenotypic correlations estimated between DIMs by RRM (Table 7) were positive except for a few estimates involving DIM 5.

## 4. Discussion

The overall mean estimated for TMY and 305MY in this study was an indicator of the performance potential of Jersey crossbreds in Tamil Nadu, as it is based on the farmers’ production system with a large dataset. The estimates of the TDMYs in the present study (Figure 1) were in the lower range compared to the values (4.70 to 10.89 kg) reported for Jersey crossbred cattle under organized farm conditions in West Bengal [18] and the values (5.41 to 10.71 kg) reported for first lactation of crossbred cattle in Kerala under field conditions [19]. The peak TDMY was reported in the TDMY1 for Jersey crossbred cattle in West Bengal [18], which then declined till the end of lactation.

The earlier studies in Tamil Nadu reported lower mean values than in this study for TMY and 305MY in Jersey × Red Sindhi [20], Jersey × Sahiwal [21], and Jersey × Tharparkar [22] crossbred cattle, and for TMY in Jersey crossbred cattle [23] in organized farms while comparable values were reported under farmers’ rearing environment [24]. Higher values were reported for TMY in Jersey × Tharparkar (or) Red Sindhi crossbreds in West Bengal [25,26] and in Jersey × Sahiwal crossbred cattle in Uttarakhand [27,28]. Several researchers reported estimates lower than the present study for both TMY and 305MY in Jersey × Red Sindhi crosses in Himachal Pradesh [29,30], for TMY in Jersey × Sahiwal crossbreds in Maharashtra [31], and Jersey × Red Sindhi in Uttarakhand [32]. The difference in the mean yields between the present study and earlier reports could be due to varying levels of Jersey inheritance, differences in rearing environments, and the fact that many of the studies took place in subtropical environments. The superior performance of Jersey crossbreds in the present study could be considered a testimony to the adaptability of the genetic group to conditions prevailing in Tamil Nadu. Hence, the advocated breeding policy [33] of crossing Jersey in the plains of the state is vindicated and should be continued in the future to increase milk production.

### 4.1. Variance Components

The initial increase in the magnitude of V_A_ followed by a decrease till the end of lactation observed in the present study for TDMYs was also reported in Sahiwal cattle in Kenya [34] and in Karan Fries cattle in India [35]. Though the study is based on the farmers’ production system, the prevailing common environmental effects at the beginning and end of lactation could be the reason for high V_EP_ under RRM. Higher V_EP_ at the beginning and at the end of lactation was also reported in primiparous Holstein cattle in Brazil [36] and in primiparous Holstein Friesian cows in Ethiopia [37]. In the present study, high estimates of V_A_ and V_P_ for TMY and 305MY were observed.

### 4.2. Heritability

The heritability estimates in mid-lactation were comparatively lower than those at the beginning and end of lactation, which may be attributed to the relatively larger V_EP_. The higher estimates of heritability observed for TDMYs at the extremes of lactation might be due to relatively higher contribution from additive action of genes to phenotypic variation. Overall, the estimates of heritability obtained in this study for TDMYs were moderate. The study is based on farmers’ production systems with several herds of small size in a progeny testing scheme. Moreover, the heterogenous nature of such a smallholder population resulted in more variation due to genes and limited relationships traced from the pedigree through the Numerator Relationship Matrix (NRM) during analyses. This results in moderate estimates of additive genetic variance and, consequently, moderate heritability. The heritability estimates for TDMYs obtained using the multivariate animal model were lower than the estimates (0.42 to 0.60) reported earlier in Jersey crossbred cattle [18]. However, the estimates were higher than those reported in Sahiwal cattle, which ranged from 0.25 to 0.30 [34], and in crossbred cattle (0 to 0.36) [19]. The higher values of heritability estimated by RRM for DIMs at the extremes of lactation (DIM 275 and DIM 305) might be due to the fact that the Legendre polynomials tend to produce variations in the extreme ends of the curve they fit. The estimates of heritability obtained using RRM in this study were higher than in the reports on Karan Fries cattle (0.17 to 0.29) [35]. For TMY and 305MY, similar estimates of heritability were reported for Jersey crossbred cattle in West Bengal [26,38] and in Tamil Nadu [25]. However, heritability estimates observed in this study were higher than those reported in Jersey crossbred cattle reared in organized farms [20,21,27,29,32].

The heritability estimates obtained in this study for TDMYs, TMY, and 305MY in Jersey crossbred cattle indicate the presence of genetic potential for these traits, which could be improved through selective breeding under farmers’ production systems in Tamil Nadu. A genetic variation identified in a population, as in the present study, indicates the advantage of a field progeny testing program compared to the usual farm-based genetic evaluations, where the population size is small, and the scope of improvement is also less. Nevertheless, in such field studies, the nature of data is unique in that relationships among animals could go unexplored due to the availability of data from a lesser number of generations. Continuing the field progeny testing program would enhance the response to selection, which is ultimately dependent on the heritability of the trait.

### 4.3. Genetic and Phenotypic Correlations

The genetic correlations of TDMYs with TMY and 305MY from multivariate animal model estimation were large and positive, indicating that the selection of animals based on early TDMYs for TMY and 305MY would be efficient. The high genetic correlation between TDMYs and 305MY found in this study agreed with the reports on Sahiwal cattle [39] and Karan Fries cattle [40]. In RRM, it was observed that the magnitude of genetic correlations decreased with an increase in the interval between the DIMs. This heterogeneous and correlated covariance structure suggests that mixed models or RRM are appropriate for the analysis of the data.

Overall, the genetic correlations estimated between TDMYs using a multivariate animal model decreased as the interval between test-days increased, consistent with the findings of [18] in Jersey crossbred cattle under organized farm conditions in West Bengal. Similar to the present study using RRM, negative genetic correlations were reported between TDMY1 and TDMY7 to TDMY9 in Karan Fries cows [35]. Additionally, the higher genetic correlations observed between DIMs that were closer in time were consistent with findings in Karan Fries cattle [35] and Sahiwal cattle [39]. The strong genetic correlations between TDMYs found in Jersey crossbred cattle in this study indicate a significant association between the additive genetic values of the traits studied. Moreover, the large positive genetic correlations between TDMYs during early lactation and TMY and 305MY suggest that selecting cows based on early TDMYs could improve TMY and 305MY.

Large positive phenotypic correlations and a trend similar to that of the present study were observed in Jersey crossbred cattle in West Bengal [18]. The decreasing estimates of phenotypic correlations with an increase in test-day interval might be attributed to the decrease in the association between traits at different stages of lactation.

## 5. Conclusions

The performance of Jersey crossbred cattle observed in the present study under farmers’ production conditions was comparable to, or even better than, in previous reports, indicating the suitability of Jersey crossbreds in the state. These results support the continued use of Jersey crossing to enhance milk production. The study favors the use of the test-day model on account of ease in data recording and also offers a successful alternative for genetic evaluation under farmers’ production systems, where recording the entire lactation of a large number of animals is almost impossible. The genetic variability for milk production was high in the farmers’ production system, and the heritabilities obtained in this study offer ample scope for improvement of milk production traits through selective breeding. The high genetic correlations observed between the TDMYs (in early lactation) and total lactation milk yield favor an early selection of cows. The documentation of this study based on a large dataset under farmers’ production system is important as very few studies on such datasets are available. Apart from genetic evaluation of the production traits, the study may be further extended by including the milk quality and functional traits to unravel the association among them for the improvement of crossbred dairy cattle in the region.

## Figures and Tables

**Table 1:** Pedigree details of Jersey crossbred cows under farmers’ production system.

Total number of animals	75,627
Number of sires with progeny record	382
Number of animals with known sire	24,158
Average number of progeny per sire	63.24
Number of animals with known dam	13,017
Number of animals with unknown dam	62,610
Number of dams with progeny record	11,785

**Table 2:** Data structure of performance records of Jersey crossbred cows under farmers’ production system.

Fixed Factor	Sub-Class	No. of Records
Agroclimatic zone	North Eastern	31,099
	North Western	15,607
	Western	25,266
	Cauvery Delta	3317
	Southern	338
Period of calving	Period 1 (2012 to 2015)	26,623
	Period 2 (2016 to 2019)	31,853
	Period 3 (2020 to 2022)	17,151
Season of calving	Winter	13,062
	Summer	22,124
	Southwest Monsoon	24,072
	Northeast Monsoon	16,369
Parity	First	36,014
	Second	15,383
	Third	11,298
	Fourth and above	12,932

**Table 3:** Descriptive statistics of test-day milk yields (kg) of Jersey crossbred cows under farmers’ production system.

Trait	*n*	Min	Max	Mean	SD	CV (%)
TDMY1	75,627	1.40	16.88	8.93	2.47	27.64
TDMY2	75,259	1.62	17.49	9.49	2.60	27.34
TDMY3	74,880	1.60	18.04	9.52	2.79	29.34
TDMY4	74,471	1.47	18.14	9.28	2.93	31.59
TDMY5	74,051	1.31	17.56	8.82	2.89	32.77
TDMY6	73,474	1.03	16.43	8.20	2.71	33.01
TDMY7	72,696	0.72	15.00	7.46	2.46	32.98
TDMY8	69,720	0.72	13.42	6.63	2.20	33.16
TDMY9	65,752	0.61	11.85	5.67	1.97	34.75
TDMY10	57,767	0.53	10.42	4.63	1.83	39.54

TDMY—Test-day milk yield, *n*—number of records, Min—minimum, Max—maximum, SD—standard deviation, CV—coefficient of variation.

**Table 4:** Estimates of variance components for test-day milk yields (TDMYs), total lactation milk yield (TMY), and 305-day milk yield (305MY) of Jersey crossbred cattle from multivariate animal model.

Trait	Additive Variance (V_A_)	Residual Variance (V_E_)	Phenotypic Variance (V_P_)
TDMY1	1.63 (0.11)	2.85 (0.08)	4.48 (0.04)
TDMY2	1.66 (0.11)	3.09 (0.09)	4.75 (0.05)
TDMY3	1.65 (0.11)	3.18 (0.09)	4.83 (0.05)
TDMY4	1.46 (0.10)	3.33 (0.08)	4.79 (0.05)
TDMY5	1.38 (0.10)	3.30 (0.08)	4.68 (0.04)
TDMY6	1.16 (0.09)	3.20 (0.07)	4.36 (0.04)
TDMY7	1.05 (0.08)	2.96 (0.07)	4.01 (0.04)
TDMY8	1.12 (0.08)	2.61 (0.06)	3.73 (0.04)
TDMY9	1.17 (0.08)	2.28 (0.06)	3.45 (0.03)
TDMY10	1.18 (0.08)	2.03 (0.06)	3.20 (0.03)
TMY	115,375 (6855)	151,756 (5408)	267,131 (2708)
305MY	111,588 (6683)	150,963 (5284)	262,552 (2650)

Figures within the parentheses indicate the Standard Error.

**Table 5:** Estimates of variance components for milk yield on different days (DIMs) of Jersey crossbred cattle from random regression model.

DIM	Additive Variance (V_A_)	Permanent Environmental Variance (V_EP_)	Residual Variance (V_E_)	Phenotypic Variance (V_P_)
5	6.34 (0.27)	4.57 (0.23)	2.42 (0.01)	13.33 (0.11)
35	2.66 (0.09)	2.34 (0.08)	2.42 (0.01)	7.41 (0.04)
65	1.69 (0.05)	1.73 (0.05)	2.42 (0.01)	5.84 (0.02)
95	2.00 (0.06)	1.99 (0.06)	2.42 (0.01)	6.41 (0.02)
125	2.61 (0.10)	2.55 (0.09)	2.42 (0.01)	7.57 (0.04)
155	3.03 (0.12)	3.04 (0.11)	2.42 (0.01)	8.48 (0.05)
185	3.23 (0.14)	3.33(0.13)	2.42 (0.01)	8.98 (0.05)
215	3.68 (0.17)	3.48 (0.15)	2.42 (0.01)	9.58 (0.06)
245	5.32 (0.23)	3.75 (0.20)	2.42 (0.01)	11.49 (0.09)
275	9.55 (0.40)	4.63 (0.34)	2.42 (0.01)	16.60 (0.16)
305	18.27 (0.74)	6.81 (0.63)	2.42 (0.01)	27.49 (0.31)

Figures within the parentheses indicate the Standard Error.

**Table 6:** Estimates of heritability (diagonal), genetic (below the diagonal), and phenotypic (above the diagonal) correlations between TDMY, TMY, and 305MY in Jersey crossbred cattle from multivariate animal model.

Trait	TDMY1	TDMY2	TDMY3	TDMY4	TDMY5	TDMY6	TDMY7	TDMY8	TDMY9	TDMY 10	TMY	MY305
TDMY1	0.36 (0.02)	0.79 (0.00)	0.68 (0.00)	0.58 (0.00)	0.51 (0.01)	0.47 (0.01)	0.44 (0.01)	0.43 (0.01)	0.40 (0.01)	0.36 (0.01)	0.67 (0.00)	0.67 (0.00)
TDMY2	0.98 (0.01)	0.35 (0.02)	0.85 (0.00)	0.74 (0.00)	0.65 (0.00)	0.59 (0.00)	0.55 (0.01)	0.52 (0.01)	0.47 (0.01)	0.41 (0.01)	0.77 (0.00)	0.78 (0.00)
TDMY3	0.95 (0.01)	0.98 (0.00)	0.34 (0.02)	0.86 (0.00)	0.76 (0.00)	0.69 (0.00)	0.63 (0.00)	0.58 (0.00)	0.51 (0.01)	0.43 (0.01)	0.82 (0.00)	0.83 (0.00)
TDMY4	0.87 (0.02)	0.93 (0.01)	0.98 (0.00)	0.30 (0.02)	0.87 (0.00)	0.79 (0.00)	0.71 (0.00)	0.64 (0.00)	0.55 (0.01)	0.45 (0.01)	0.84 (0.00)	0.85 (0.00)
TDMY5	0.81 (0.02)	0.88 (0.02)	0.94 (0.01)	0.99 (0.00)	0.29 (0.02)	0.88 (0.00)	0.79 (0.00)	0.71 (0.00)	0.60 (0.00)	0.49 (0.01)	0.85 (0.00)	0.85 (0.00)
TDMY6	0.78 (0.02)	0.85 (0.02)	0.91 (0.01)	0.97 (0.01)	0.99 (0.00)	0.27 (0.02)	0.88 (0.00)	0.78 (0.00)	0.67 (0.00)	0.55 (0.01)	0.85 (0.00)	0.85 (0.00)
TDMY7	0.75 (0.03)	0.81 (0.02)	0.88 (0.02)	0.95 (0.01)	0.97 (0.01)	0.99 (0.00)	0.26 (0.02)	0.86 (0.00)	0.75 (0.00)	0.62 (0.00)	0.83 (0.00)	0.84 (0.00)
TDMY8	0.77 (0.03)	0.81 (0.02)	0.86 (0.02)	0.91 (0.01)	0.93 (0.01)	0.95 (0.01)	0.98 (0.00)	0.30 (0.02)	0.86 (0.00)	0.73 (0.00)	0.83 (0.00)	0.83 (0.00)
TDMY9	0.76 (0.03)	0.79 (0.02)	0.82 (0.02)	0.86 (0.02)	0.87 (0.02)	0.89 (0.02)	0.93 (0.01)	0.98 (0.00)	0.34 (0.02)	0.86 (0.00)	0.78 (0.00)	0.78 (0.00)
TDMY10	0.75 (0.03)	0.75 (0.03)	0.76 (0.03)	0.76 (0.03)	0.76 (0.03)	0.79 (0.02)	0.84 (0.02)	0.93 (0.01)	0.98 (0.00)	0.37 (0.02)	0.70 (0.00)	0.69 (0.00)
TMY	0.90 (0.01)	0.93 (0.01)	0.96 (0.01)	0.96 (0.01)	0.95 (0.01)	0.95 (0.01)	0.95 (0.01)	0.96 (0.01)	0.95 (0.01)	0.90 (0.01)	0.43 (0.02)	0.99 (0.00)
305MY	0.90 (0.01)	0.94 (0.01)	0.96 (0.01)	0.97 (0.01)	0.96 (0.01)	0.95 (0.01)	0.95 (0.01)	0.96 (0.01)	0.94 (0.01)	0.89 (0.01)	1.00 (0.00)	0.43 (0.02)

Figures within the parentheses indicate the Standard Error.

**Table 7:** Estimates of heritability (diagonal), genetic (below the diagonal) and phenotypic (above the diagonal) correlations between DIMs of Jersey crossbreds from random regression model.

Milk Yield on Day	5	35	65	95	125	155	185	215	245	275	305
5	0.48 (0.02)	0.69 (0.00)	0.40 (0.00)	0.12 (0.00)	−0.05 (0.00)	−0.12 (0.01)	−0.11 (0.01)	−0.02 (0.01)	0.12 (0.01)	0.27 (0.01)	0.39 (0.01)
35	0.93 (0.00)	0.36 (0.01)	0.53 (0.00)	0.34 (0.00)	0.21 (0.00)	0.14 (0.00)	0.13 (0.00)	0.15 (0.00)	0.20 (0.00)	0.26 (0.00)	0.29 (0.01)
65	0.56 (0.02)	0.83 (0.01)	0.29 (0.01)	0.54 (0.00)	0.48 (0.00)	0.43 (0.00)	0.40 (0.00)	0.36 (0.00)	0.30 (0.00)	0.22 (0.00)	0.15 (0.00)
95	0.12 (0.03)	0.48 (0.02)	0.89 (0.01)	0.31 (0.01)	0.63 (0.00)	0.61 (0.00)	0.57 (0.00)	0.50 (0.00)	0.37 (0.00)	0.21 (0.00)	0.07 (0.00)
125	−0.11 (0.03)	0.26 (0.03)	0.75 (0.01)	0.97 (0.00)	0.35 (0.01)	0.69 (0.00)	0.66 (0.00)	0.58 (0.00)	0.43 (0.00)	0.24 (0.00)	0.07 (0.01)
155	−0.16 (0.03)	0.19 (0.03)	0.67 (0.02)	0.92 (0.00)	0.99 (0.00)	0.36 (0.01)	0.71 (0.00)	0.65 (0.00)	0.51 (0.00)	0.32 (0.01)	0.14 (0.01)
185	−0.09 (0.03)	0.19 (0.03)	0.63 (0.02)	0.85 (0.01)	0.92 (0.00)	0.97 (0.00)	0.36 (0.02)	0.71 (0.00)	0.62 (0.00)	0.45 (0.00)	0.29 (0.01)
215	0.09 (0.03)	0.21 (0.03)	0.57 (0.02)	0.70 (0.01)	0.76 (0.01)	0.84 (0.01)	0.94 (0.00)	0.39 (0.02)	0.72 (0.00)	0.62 (0.00)	0.49 (0.00)
245	0.32 (0.03)	0.40 (0.03)	0.46 (0.02)	0.45 (0.02)	0.48 (0.02)	0.58 (0.02)	0.75 (0.01)	0.93 (0.00)	0.46 (0.02)	0.78 (0.00)	0.71 (0.00)
275	0.49 (0.03)	0.46 (0.02)	0.33 (0.02)	0.20 (0.02)	0.20 (0.03)	0.30 (0.03)	0.50 (0.02)	0.76 (0.01)	0.95 (0.00)	0.58 (0.02)	0.85 (0.00)
305	0.59 (0.02)	0.48 (0.02)	0.23 (0.02)	0.02 (0.02)	−0.01 (0.03)	0.09 (0.03)	0.30 (0.03)	0.60 (0.01)	0.86 (0.01)	0.98 (0.00)	0.67 (0.02)

Figures within the parentheses indicate the Standard Error.

## Data Availability

3rd Party Data. Restrictions apply to the availability of these data. Data were obtained from the Tamil Nadu Co-operative Milk Producers’ Federation (TCMPF), Chennai, India, and are available with permission from the TCMPF.
